# Tuberculosis Knowledge, Attitude, and Practice (TB-KAP) among the volunteers: Findings from community-based intervention training outcome in Tribal Hard-to-Reach Areas in Manipur

**DOI:** 10.1371/journal.pone.0356469

**Published:** 2026-08-14

**Authors:** Karikalan Nagarajan, Stephen Arangba, Basilea Watson, Muniyandi Malaisamy, Linette Muanching, Suchita Singh, Sanjay Kumar Mattoo, Vijaya Elangbam, W. Shashi Singh, Senthil Sellappan, Dahiiru Ngade, Amow Ngaopuo, Lungnalii K. T, Thangpa Serto, Pfokreho Pfoze, Dina Nair, Vignes Anand Srinivasalu, Elizabeth R K, Pung Mark, Hanna R N, Peter Yonuo, Sumpi Percy, Harpreet Kaur

**Affiliations:** 1 ICMR-National Institute for Research in Tuberculosis, Chennai, India; 2 National TB Elimination Programme, Imphal, Manipur, India; 3 Indian Council of Medical Research Headquarters, New Delhi, India; 4 Central TB Division, New Delhi, India; 5 Regional Institute of Medical Science, Imphal, Manipur, India; 6 Directorate of Health Services, Imphal, Manipur, India; 7 National Health Mission, Senapati, Manipur, India; 8 Senapati District Student Association, Senapati, Manipur, India; 9 Senapati District Women Association, Senapati, Manipur, India; National Institute of Tuberculosis and Respiratory Diseases, INDIA

## Abstract

**Background:**

Involving community volunteers in the tuberculosis (TB) program is widely acknowledged and considered a key element of the community engagement strategy in the TB program of India, especially in predominantly tribal North Eastern districts. Inadequate TB knowledge, attitudes and Practices (KAPs) about health and related risk factors could be a key barrier to using community volunteers or lay persons in health program.

**Methods:**

We trained 708 volunteers from student and women organisation (SAWOs) in 189 villages, Senapati District, Manipur between February 2023 and August 2025. A training on identifying TB symptomatics, sputum collections, transportations, patient support and follow-up during their treatment, counselling were provided. A TB-KAP pre and post data were collected in REDCap software and analysed using Stata. To assess the impact of the training on participants’ KAP regarding TB, both descriptive and inferential statistical methods were used. Statistical significance was determined at *p-value* <0.05.

**Results:**

Of 708 trained volunteers, 339 were retained during the full training and assessment. With respect to the knowledge score, the probability of being in the highest knowledge category increased from 10% to 65% after training, while the likelihood of being in the lowest two categories dropped from 61% to 8%. The mean attitude score about TB increased from 24.53 (SD = 4.79) to 25.40 (SD = 4.83) after training. This difference of 0.87 points was statistically significant (*t*(353)=−3.58, *p* = 0.0004), with a 95% confidence interval of [−1.35, −0.39]. Significant positive changes were seen in the anticipated health-seeking intentions of the volunteers in terms of seeking care for symptoms within 1–2 weeks (*p* = 0.0009).

**Conclusion:**

Our study demonstrated that focused and culturally appropriate short term training can produce significant immediate gains in TB knowledge, with modest improvements in attitudes and reported health-seeking intentions, even in the remotest tribal areas among community volunteers.

## Introduction

India accounts for an estimated 2.1 million new Tuberculosis (TB) cases in 2024, making it the country with the highest incidence of TB worldwide [1]. Despite providing free diagnosis and treatment under the National TB Elimination Program (NTEP), delays in care seeking, underreporting, and poor treatment adherence remain a critical issue, especially in tribal areas. In India, most tribal populations live in remote areas and reside in scattered habitats, have a low level of literacy, wealth, and awareness, and have a deep rooted belief in traditional healers and are heavily reliant on the traditional self preparing medicine (herbal) to treat themselves [2]. Their access to health services is severely restricted by a variety of factors shaped by deep-rooted socio-cultural, geographical, economic and systemic factors [3,4].

Most tribal populations with TB or at risk of TB do not have access to TB screening and diagnostic services in time, which delays treatment initiation [5]. For this, the NTEP had identified key vulnerable groups that suffer a high burden of TB or are at high risk of TB, including tribal populations, to carry out Active Case Finding (ACF). However, to carry out ACF activities in most tribal areas remains a huge challenge for the TB program due to their remote settlement, difficult terrain, resource constraints, and inadequately trained health care professionals [6–8]. To overcome these barriers, NTEP had emphasised community driven approaches for improving case detection, treatment and care among tribal populations^10^. Involving community volunteers in the TB program is widely acknowledged and considered a key element of the community engagement strategy in the TB program of India, especially in predominantly tribal districts [9]. However, research has shown that inadequate knowledge and wrong perceptions and attitudes about health and related risk factors could be a key barrier to using community volunteers or lay persons in health programs [10–14]. Evidence has shown that correct knowledge, attitude and practice are important drivers for the effective involvement of any volunteer in any health care program, including the TB program [15–17]. The World Health Organisation also recommends that community volunteers need to be provided with appropriate training to ensure that community-centred approaches effectively support healthcare programs [18–20]. In this context, there is a need to provide training to community volunteers who will be involved in health programs.

While several past initiatives involved community volunteers for TB in India [21–28]. There is a dearth of information on what, how, who, and where the training was provided. Also, there dearth of information providing training to the organised students and women’s organisations from the tribal community in North East India, a predominantly tribal and hard-to-reach region. Moreover, evidence is limited with regard to the provision of TB related training for tribal community volunteers, especially from particularly vulnerable tribal groups (PVTGs) in North East India and the outcomes of such training.

Therefore, this study aims to assess the outcomes of a two day tailored training program (hands-on training) imparted to tribal TB volunteers drawn from organised student and women organisations from the hard-to-reach tribal dominated Senapati district in Manipur before implementing a larger community based intervention program, in terms of their TB-related KAP.

## Methodology

This study is part of a larger quasi experimental pre and post study, which has been carried out between February 2023 to August 2025 in the whole District of Senapati, Manipur. The present sub study conducted among students and members (≥18 years) of women organisations (SAWOs) in Senapati, was aimed at assessing the impact of engaging them in improving TB case detection, treatment adherence and treatment outcomes as compared to existing routine NTEP strategies. The study has been implemented in two phases: (1) Preparatory and (2) Intervention phase. During the preparatory phase, rapport building, volunteer recruitment, volunteers’ training, assessing pre and post TB knowledge, attitude and practice, TB awareness campaign, village mapping, household enumeration and TB enhanced case finding activities were carried out. In the intervention phase, two rounds of door-to-door TB active case finding, helping in sample transportation and supporting the patients during their treatment activities were carried out. The detailed study methodology has been explained elsewhere [29].

### Volunteers sample size

As per the NTEP Senapati report, there are 189 villages, 47,411 households and population of 285,404 in Senapati district, Manipur. We assume that one volunteer can complete the assigned project activities of 150 (maximum) households in a specific project period, so we have recruited one volunteer per 150 households in each village. Hence, totally, our target was to recruit 385 volunteers from 189 villages in Senapati District, Manipur, to carry out the activities. A village where the total household was less than 150 households was also considered to recruit one volunteer per village, considering the difficult terrain, distance, language barriers, and social acceptability of an outsider by the native villagers.

### Volunteer recruitment

The volunteers were recruited through SAWOs at the village level based on the number of households present in each village. A list of nominated individuals who are willing to become volunteers was received from the SAWOs. From the nominated list, the volunteers were selected based on the number of volunteers required in each village and based on their eligibility to become a volunteer as per our study protocol.

### Eligibility criteria

The eligibility criteria to become a volunteer are (1) ≥18 years, (2) members of local SAWOs, (3) literate and fluent in the local language, (4) willing to undergo two days of hands-on training, (5) committed to be in the local areas where they are residing for a minimum of one year, (6) provide written consent and (7) not diagnosed with active TB at the time of enrolment.

### Volunteers training

The eligible and selected volunteers underwent two days of hands-on training using a specially tailored training manual developed by the study team for the project. The training was provided by an expert team consisting of public health experts, medical officers, sociologists, social workers, biostatisticians, health economists, and laboratory experts. The training syllabus includes the basics of TB, identifying presumptive TB based on symptoms, sputum collections and transportations, importance of patient support, and follow-up during their treatment, basic counselling principles, data collection process and tools, and ensuring understanding of the study protocol. All the trainings were conducted in batches on different dates and time, consisting of a maximum of 30 volunteers at a time at the District Tuberculosis Centre (DTC), Senapati, Manipur. During the training period, TB-KAP assessment was conducted by the trainers (study team) before and after completing the training to ensure that the volunteers are equipped with adequate knowledge about the basics of TB before starting the field activities. Additionally, one day of training was provided to the volunteers who underperformed in our assessment. The additional one-day training was provided either in the DTC, Senapati or at the field level based on the volunteers’ convenience

### Data collection

TB-KAP pre- and post-data were collected in REDCap software (an electronic data capturing tool) hosted at ICMR-NIRT. The baseline data was collected on the first day before providing the training, post obtaining written consent. Endline data was collected on the second day post completion of the training. A REDCap link was shared with the volunteers via WhatsApp before starting the training, and after completion of the training, to fill out the questionnaire.

### Tools of data collection

A standardized, pretested questionnaire adapted from WHO guideline for conducting TB KAP studies was used to collect data [30]. The questionnaire was pilot-tested with a small group of local health staff prior to field use to assess clarity and cultural appropriateness. The internal consistency of the knowledge and attitude scales was assessed using Cronbach’s alpha. The TB-KAP questionnaire consists of socio-demographic details, TB-KAP related questions. Totally, there were 30 questions apart from socio-demographic details. TB knowledge questions consist of 15 questions, 8 attitude questions and 4 questions related to practice

### Data management

All the data were managed using REDCap software and were stored securely at ICMR-NIRT. REDCap is a web based application designed to support data capture for research studies. It provided an intuitive interface for validated data entry, audit trails for tracking data manipulation. REDCap implements authentication to validate the identity of end users, which allows only users with proper privileges to utilize the system. The REDCap system has inbuilt data validation and checks in order to ensure the consistency of the data. Also, regular quality control of the data was done in order to ensure valid data collection.

### Ethical consideration

The institutional ethics committee approval was obtained from the Indian Council of Medical Research, National Institute for Research in Tuberculosis (ICMR-NIRT) registration No.234/NIRT-IEC/2022 and Regional Institute of Medical Science (RIMS) registration No.A/206/REB/Prop(FP)186/114/25/2022. The institution’s ethics committee approved participant information sheet and consent form were used to obtain consent from the volunteers before undergoing the training.

### Statistical analysis

Data analysis was conducted using Stata 17.0. To assess the impact of the training on participants’ KAP regarding TB, both descriptive and inferential statistical methods were used. Knowledge score, with a maximum score of 25 and a minimum of 0 was developed based on 15 knowledge questions. The score was a composite count of all correct responses: single-correct items each contributed one point, while multiple-response (“select all that apply”) items contributed one point for each correct option selected (up to nine points for symptoms, two for diagnostic tests, and five for risk factors), giving a total possible score of 0–25. Knowledge score was categorised as very low (0–5), low (6–10), moderate (11–15), or high (16–25). For TB knowledge (ordinal outcome), a mixed effects ordinal logistic regression (meologit) was performed with a random intercept at the participant level (identified by Mobile No.) to account for repeated measurements before and after training. Independent variables included demographic characteristics such as age, gender, marital status, education and occupation. Odds ratios (ORs) with 95% confidence intervals (CIs) were also reported.

The KAP questionnaire consisted of 8 statements on attitude. The responses were of a Likert scale (1 is Strongly Disagree and 5 is Strongly Agree). The score for the negative statements was reversed. A score was calculated by summing up these 8 statements. Several of these statements captured stigma-related dimensions (e.g., desire to help, avoiding or keeping away from a person with TB, fear, embarrassment or shame, and rejection); negatively worded stigmatising items were reverse-scored so that higher scores reflect less stigmatising, more favourable attitudes. The score had a minimum value of 8 and a maximum of 40. The average score before and after the training was determined using the mean and standard deviation.

The attitude score was divided into three groups. The distribution of these scores was compared before and after training. No significant difference was observed in the score. To evaluate changes in attitude scores, a paired t-test was used, comparing mean attitude scores before and after training. For categorical attitude variables with more than two levels, the Stuart-Maxwell test of marginal homogeneity was applied to assess shifts across categories while accounting for paired responses. For categorical knowledge and practice items (e.g., perceived seriousness of TB, help-seeking behaviour), McNemar’s chi-square test was used to assess pre and post training differences for binary variables. For nominal variables with more than two categories, the symmetry test was applied. No formal correction for multiple comparisons was applied; findings should be interpreted in the context of the exploratory nature of the sub-analyses. Statistical significance was determined at *p-value* <0.05.

## Results

**Fig 1:**
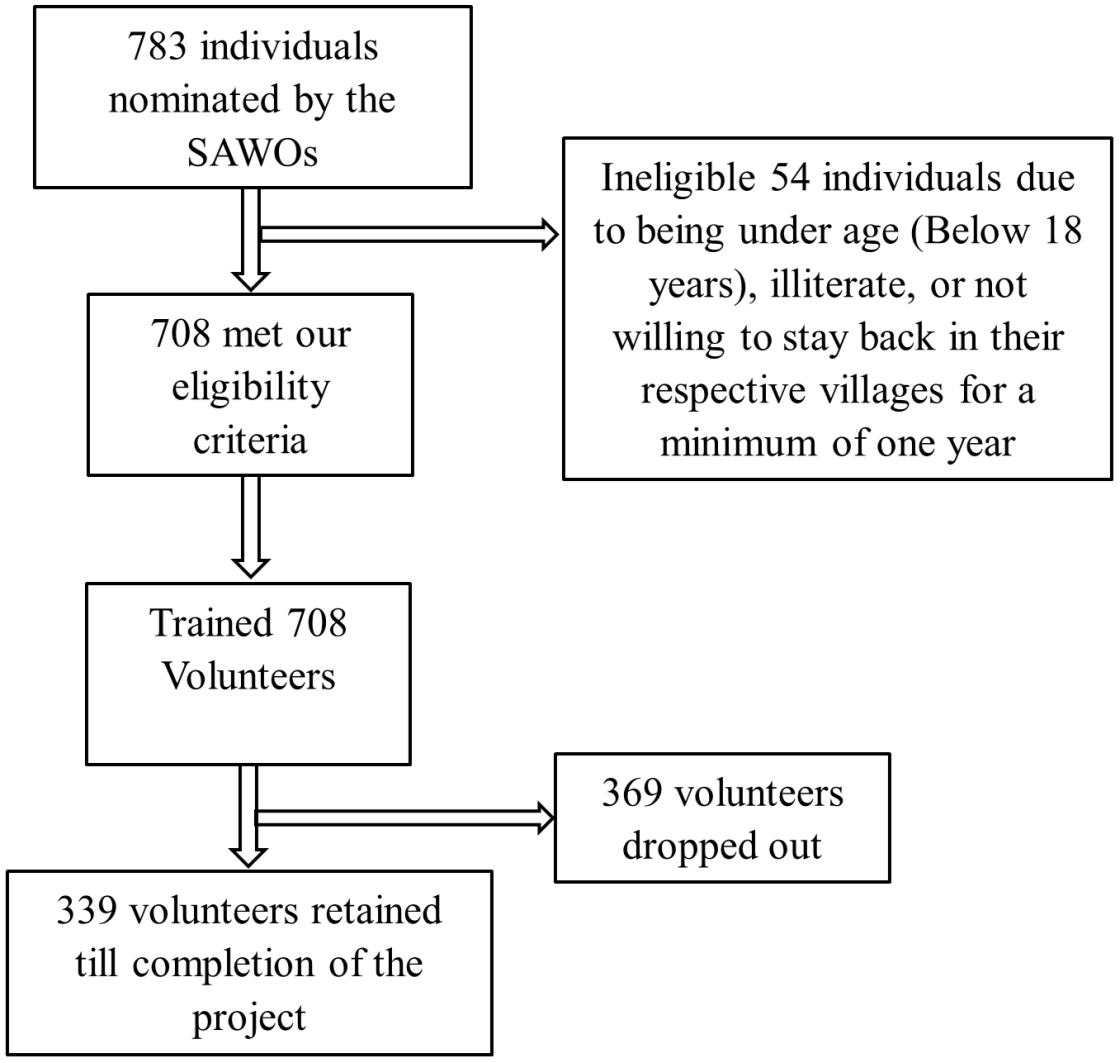
Volunteer recruitment and retention.

Of the 783 individuals nominated by the SAWOs who are willing to become volunteers from all the villages, 54 individuals were ineligible because of being under age (<18 years), illiterate, or not willing to stay back in their respective villages for a minimum of one year. Of the 783 nominated and willing individuals, 708 met our eligibility criteria. All 708 individuals provided their consent and underwent training. Of the 708 trained volunteers, 369 dropped out at different stages and times. The common reasons for dropping out were low incentive, not being able to spare time, moving out of town for job/studies and a few have expressed not being interested anymore in volunteering. Of the 708 trained volunteers, 339 remain active till completion of the project (Fig 1). Of the 708 trained volunteers, 480 had filled the baseline questionnaire and 424 had filled the endline questionnaire. Out of 708 trained volunteers, 408 have filled out both pre and post TB-KAP assessment questionnaires.

### Participant characteristics

Of the 424 volunteers who attended the baseline training (Table 1). Almost one third (31%) of the volunteers were from the Mao block, and more than half (53%) of them were male. Majority (83%) of volunteers were <34 years of age, unmarried (73%) and had an education level up to higher secondary or graduation (64.4%). More than one third (35%) of the volunteers were students. Majority (86%) of the volunteers earned less than ₹10000 and were predominantly (98%) from scheduled tribes.

**Table 1:** Socio-demographic profiles of volunteers (n = 424, baseline completers).

	Categories	No	%
**Block**	Mao	147	31
	Paomata	84	18
	Oinam	95	20
	Maram	103	21
	Senapati HQ	50	10
**Age**	18-24 years	200	42
	25-34 years	200	42
	35-44 years	58	12
	45-55 years	22	5
**Gender**	Male	256	53
	Female	224	47
**Marital status**	Married	132	27
	Unmarried	348	73
**Education**	No formal school	7	2
	Upto primary level	44	9
	Upto secondary level	97	20
	Upto higher secondary level	139	29
	Upto graduation/degree level	193	40
**Occupation**	Housewife	67	14.0
	Student	167	34.8
	Own cultivation	120	25.0
	Agriculture labourer	41	8.5
	Small business/petti/ tea shop	28	5.8
	Livestock	2	0.4
	Private	68	14.2
	Government	7	1.5
	Other	32	6.7
**Monthly income**	No income	225	47
	Up to ₹10000	188	39
	>₹10001	67	14
**Schedule tribe**	Yes	472	98
	No	8	2

### Volunteers’ knowledge score

**Fig 2:**
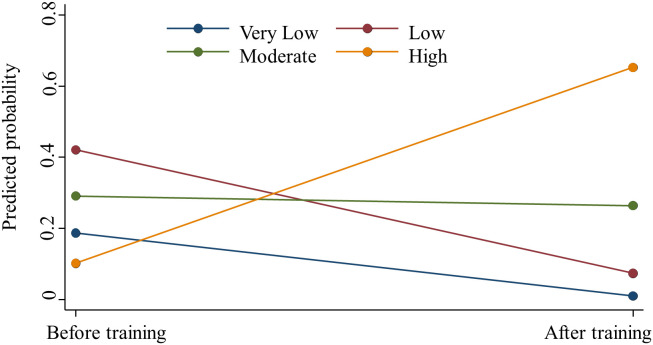
Predicted probabilities of TB knowledge before and after training.

With respect to the knowledge score (out of 25), which was computed before and after the training, the majority of the participants had low and moderate knowledge scores before the training, while the majority had higher knowledge scores after the training. A mixed effects ordinal logistic regression was used to evaluate changes in TB knowledge before and after training, adjusting for repeated measures on individuals. The model showed a statistically significant and substantial improvement in knowledge scores following training (OR=29.4, 95% CI: 18.9–45.9, p < 0.001). While this OR appears large, it is consistent with the magnitude of the observed shift: the predicted probability of being in the highest knowledge category increased from 10% to 65%, and such shifts in cumulative log-odds are expected to produce large ORs in ordinal models. The random intercept for participant ID significantly improved model fit (χ² = 17.80, p < 0.001), confirming the importance of adjusting for within subject pairing (Fig 2).

### Volunteers’ knowledge score and socio-demographic variables

The univariate ordinal logistic regression shows that participants aged 25–34 years had significantly higher odds of better TB knowledge compared to those aged 18–24 years (OR=1.7; 95% CI: 1.19–2.47; p = 0.00), while no significant difference was observed in the 35–44 age group. Females were more likely to have higher TB knowledge than males (OR=1.5; 95% CI: 1.11–2.15; p = 0.01). Marital status did not show a significant association with TB knowledge. Regarding education, only those with graduation level education had significantly higher knowledge compared to those with primary education (OR=2.7; 95% CI: 1.5–4.7; p = 0.001), while secondary and higher secondary levels showed non-significant positive trends. In terms of occupation, individuals working in the private sector (OR=2.5; 95% CI: 1.50–4.10; p = 0.00) and government sector (OR=6.2; 95% CI: 1.41–27.34; p = 0.02) had significantly higher TB knowledge, whereas those involved in own cultivation had significantly lower knowledge (OR=0.7; 95% CI: 0.45–0.97; p = 0.04). Other occupational categories, including housewives, students, agricultural labourers and small business owners, did not show significant associations (Table 2).

**Table 2:** Knowledge score and socio-demographic variables.

Variable	Category	OR 95% CI	p-value
**Age in years**	18-24	1	
	25–34	1.7 (1.19-2.47)	0.001
	35–44	1.2 (0.74-1.89)	0.49
**Gender**	Male	1	
	Female	1.5 (1.11-2.15)	0.01
**Marital Status**	Married	1	
	Unmarried	1.2 (0.81-1.69)	0.4
**Education**	Primary	1	
	Secondary	1.4 (0.7-2.5)	0.3
	Higher secondary	1.4 (0.8-2.6)	0.21
	Graduation	2.7 (1.5-4.7)	0.001
**Occupation**	Student	1 (0.74-1.47)	0.83
	Housewife	1.2 (0.76-1.96)	0.41
	Own cultivation	0.7 (0.45-0.97)	0.04
	Agriculture labourer	0.9 (0.50-1.58)	0.68
	Small business	1.4 (0.67-2.77)	0.39
	Livestock	0.7 (0.07-7.05)	0.78
	Private	2.5 (1.50-4.10)	0.001
	Government	6.2 (1.41-27.34)	0.02
	Other	0.6 (0.29-1.14)	0.11

### Multiple ordinal logistic regression

A stepwise mixed-effects ordered logistic regression analysis was performed using meologit in Stata to identify factors associated with TB knowledge score. The dependent variable was modelled as an ordinal outcome with random intercepts for each individual to account for repeated measures or clustering. Starting with a full model including all socio-demographic predictors and occupation types, variables were removed step-by-step based on statistical significance (p < 0.05), model fit (log-likelihood), and clinical relevance. After each step, the log likelihood and Wald Chi square statistics were examined to ensure that model fit remained acceptable. The final model retained key demographic covariates (age, gender, education) and a subset of occupation categories that showed potential influence. Training status (pre vs. post) was entered as a forced variable in all model steps; stepwise selection was applied only to the socio-demographic covariates.

The final model demonstrated that post training status, female gender, age group 25–34 years, graduation level education, and working in the private sector or government sector were significantly associated with higher TB knowledge scores. The model also showed a significant random effect for the grouping variable, justifying the use of a mixed effects framework.

### Volunteers’ attitude score

A comparison of pre and post training responses revealed a significant improvement in participants’ awareness and perceptions related to TB. Prior to training, only 61% of participants considered TB to be a very serious disease. This proportion increased markedly to 79% after the training (p < 0.001). Similarly, the perception of TB as a public health problem in India rose substantially from 71% to 95% (p < 0.001), with corresponding decreases in uncertainty and denial. Notably, recognition of personal susceptibility to TB also improved, with 80% of participants post training acknowledging that they could get TB compared to 58% before training (p < 0.001).

Across all three knowledge items, the proportion of “Don’t know” responses dropped significantly after training, indicating enhanced clarity and reduced ambiguity in understanding TB. These shifts suggest that the training intervention was effective in improving TB-KAP among the participants (Table 3).

**Table 3:** Volunteers’ attitude score.

Categories	Before training	After training	p-value
	No (%)	No (%)	
How serious is TB?			<0.001
Very serious	270 (61)	333 (79)	
Somewhat serious	107 (24)	63 (15)	
Not very serious	33 (8)	23 (6)	
Don’t know	30 (7)	2 (1)	
Is TB public health problem?			<0.001
Yes	314 (71)	400 (95)	
No	12 (3)	1 (0.2)	
Maybe	76 (17)	17 (4)	
Don’t know	38 (9)	3 (1)	
Do you think you can get TB?			<0.001
Yes	253 (58)	336 (80)	
Sometime	108 (25)	58 (14)	
No	31 (7)	20 (5)	
Don’t know	47 (11)	7 (2)	

### Volunteers’ attitude mean score

A paired *t*-test was conducted to assess whether there was a significant change in participants’ attitude scores following the training. The mean attitude score increased from 24.53 (SD = 4.79) before training to 25.40 (SD = 4.83) after training. This difference of 0.87 points was statistically significant (*t*(353)=−3.58, *p* = 0.0004), with a 95% confidence interval of [−1.35, −0.39]. These results indicate a statistically significant improvement in mean attitude scores post training. However, on a scale ranging from 8 to 40 the 0.87-point change corresponds to a small effect size (Cohen’s d ≈ 0.19) and is of modest magnitude. The statistical significance therefore reflects a consistent but small upward shift rather than a large change, consistent with the categorical analysis below, in which the overall distribution of attitude categories did not change significantly.

### Volunteers’ attitude category scores

A Stuart-Maxwell test was used to assess whether participants’ attitudes significantly changed after training. The results showed no statistically significant difference in the marginal distribution of attitude categories before and after training (χ² = 0.62, df = 2, p = 0.16). Similarly, Bowker’s test of symmetry indicated no significant asymmetry in the shift patterns between attitude categories (χ² = 5.05, df = 3, p = 0.17). This suggests that while individual shifts occurred, the overall distribution of attitudes remained relatively stable post-training. The t-test detects any consistent directional movement in the continuous score, while the Stuart-Maxwell test requires sufficient movement across category thresholds, a higher bar given the small effect size (Cohen’s d ≈ 0.19).

### Volunteers’ practice-related changes

With respect to practices related to TB, significant positive changes were seen in the reported, anticipated health-seeking intentions assessed immediately after training**.** For example, more participants indicated they would talk to a doctor or family member if symptomatic. Also, there was a notable shift toward earlier care-seeking intentions, with more people willing to go to a facility within 1–2 weeks of symptom onset (*p* = 0.001). These findings support improved health-seeking intentions post-training (Table 4).

**Table 4:** Volunteers’ practice related changes before and after training.

About your illness	Before training	After training	p-value
	No (%)	No (%)	
Who would you first talk?^*^			
Doctor	336 (48)	360 (52)	<0.001
Spouse	52 (37)	89 (63)	<0.001
Parents	238 (49)	251 (51)	<0.001
Children	22 (32)	46 (68)	<0.001
Family members	31 (32)	65 (68)	<0.001
Close friend	53 (47)	59 (53)	0.07
No one	1 (50)	1 (50)	0.32
Where would you go?			0.36
Hospital	421 (51)	410 (49)	
Medical shops/pharmacy	14 (58)	10 (42)	
Traditional healer	3 (100)	0 (0)	
Others	1 (50)	1 (50)	
What point of time you go to health facility?			0.0009
When own treatment does not work	59 (69)	26 (31)	
When symptoms last for 1–2 weeks	96 (42)	133 (58)	
When symptoms last for 3–4 weeks	23 (74)	8 (26)	
When I realize I have TB symptoms	255 (50)	252 (50)	
I will not go to the Doctor	6 (75)	2 (25)	
Reason for not going to health facility			
Not sure where to go	4 (80)	1 (20)	
Distance to clinic	1 (50)	1 (50)	

* Respondents could select more than one person. McNemar’s test applied separately to each binary option.

## Discussion

In this quasi-experimental pre-post study, we have assessed the impact of two days of hands-on training among the community volunteers. The study found a significant increase in terms of TB-KAP related scores after a brief two day hands-on training. This is quite significant considering the large sample of volunteers who were trained in a short period of time. Such culturally appropriate targeted training for volunteers will be of utmost importance in tribal hard-to-reach geographies where TB-KAP are relatively poor, which in turn impacts the health seeking behaviour of the population adversely.

Our study finding shows low (60.8%) TB knowledge among the volunteers before our training intervention. However, post intervention, we observed significant improvement in TB knowledge among the volunteers (from 10–65%); also, those with low TB knowledge fell from 61–8%. Previous studies have reported mixed results regarding TB knowledge among the community in India. Some studies have reported that 70–89% of the Indian population had heard about TB [31,32], however, in-depth knowledge about the causes, mode of transmission and prevention is poor [33]. Moreover, some studies have reported low TB awareness among community members, especially in the tribal hard-to-reach areas and rural populations [4,34–36]. The study findings indicated that, in general, though the TB awareness in India reflected on the higher end, it does not truly represent those hard-to-reach tribal and rural areas. The discrepancies in findings could be attributed to differences in the target population, measurement indicators, other sociodemographic factors, lack of sustained effective coverage of IEC intervention based on region/areas, resource constraints and barriers in conducting mass media or mid media interventions. Also, it is not only about merely hearing about TB from any sources that could make a difference; what matters is having correct knowledge and understanding about the TB disease, the causes, mode of transmission, and methods of prevention are important to control and prevent further transmissions. In this regard, our intervention has shown that there is a possibility to educate and sensitise a large group of volunteers who represent various tribal communities at the village level through a culturally appropriate, comprehensive training session done in a short time frame. Our study findings indicate that this bottom-up approach could be a game changer in North Eastern India to strengthen the TB awareness and advocacy, communication and social mobilisation intervention in a consistent and sustainable way. A similar community based training intervention gain was observed in Nigeria [15], Indonesia [37], Thailand [38] and Maharashtra, India [39]. These findings indicate that our targeted education has effectively addressed, to a large extent, the gaps that we found before our intervention and emphasised the need for culturally appropriate targeted training for those unattained populations.

Our analysis also highlights sociodemographic predictors of TB knowledge. Volunteers who were between the ages of 25–34 showed a better knowledge score than the younger (adolescent and youth) and middle-aged groups, which is consistent with previous studies’ findings [40,41]. Higher education level was a significant predictor of knowledge scores among the volunteers. This is in line with previous studies linking higher education to health awareness [33,40,42]. Working in the formal sectors (either government or private) by occupation was found to be driving TB knowledge among volunteers as compared to those in agricultural and other informal sector work. These differences in knowledge level based on occupation could be attributed to social interaction, exposure to public health awareness campaigns and activities as part of their workplace mandates in the formal sector, which could drive up their TB knowledge. Previous studies have also reported a similar finding that people who are employed in the organised sector have better knowledge as compared to working in the unorganised sector [40–42]. These findings emphasise that recruiting employed volunteers under the formal sector could be an advantage in training them. However, it remains crucial to engage homebound and informal sector individuals who often dominate remote, hard-to-reach tribal villages. These counters who have higher penetration at the village and hamlet level could be important in TB elimination activities and tailored sensitisation intervention is required for them. Our study also found a gender difference in TB knowledge. Female volunteers were more odds to have better knowledge as compared to males, which is consistent with previous study findings [40]. These findings underscore women’s greater involvement in family health matters, which might explain higher TB knowledge among our female volunteers.

With regard to the volunteer attitude towards TB, it was found that the volunteers’ attitude and perception improved post training. Our study found that the volunteers’ perception of TB as a very serious health concern increased from 61–79% post training sessions. Also, recognising TB as a public health problem jumped from 71–95%. The mean attitude score increased significantly (by about 0.87 points), indicating more favourable attitudes post training. Although statistically significant, this change is small in magnitude on the 8–40 scale, and the more substantial training gains were observed for knowledge rather than attitude. This attitude shift echoes a similar study conducted in South west Nigeria in 2015. They found that volunteer led education significantly improved the community’s attitude toward TB [15]. Our findings indicated that the training content of the volunteers, which had emphasized the TB burden and elimination goals of the state and country, could have helped the volunteers understand TB from a public health perspective. Therefore, while designing a training course for the grassroots level workers, incorporating a public health perspective of the diseases from a national perspective could help, rather than focusing only on the TB-KAP. This is quite important since the motivation for volunteers stems not just from understanding about the TB but also from its consequences for society as well.

Similarly, the trend associated with the recognition of personal susceptibility to TB improved from 58–80% post training session. Previous studies have shown that knowledge alone may not result in healthy behaviour changes, but self risk perceptions hold the key to any ideal behaviour change [43,44]. It must be noted that although the attitude of the participants’ scores increased, many remained within the same category, resulting in a significant difference in means without a corresponding significant change in category distribution. This suggests that the training had a positive effect on attitude scores, even if it did not markedly shift the categorical levels. Thus, imparting risk perception related cues, information and content in the training still needs to be strengthened and provided to grassroots level TB health workers.

Importantly, our training has also impacted health seeking practice perceptions; we found that a greater number of volunteers were inclined to seek a physician and family members’ assistance if they develop TB symptoms. Moreover, the volunteers were inclined to go to health facilities within 1–2 weeks of experiencing symptoms. These findings support improved health seeking attitudes among the volunteers as a result of training. These attitudes are encouraging, provided that delayed care seeking in India is common and leads to continued transmission. As per the latest National TB prevalence survey conducted in 2019–2021, only 36% of people with presumptive TB seek health care in India [45]. Sensitising the grassroots level volunteers on the appropriate health seeking behaviours could be the most important strategy, hence this could have a multiplier effect on the health-seeking at the family and village level where these volunteers are living.

The key strength of this study lies in its quasi-experimental pre and post design, which allows us to assess the impact of training outcomes immediately. Also, the study has especially focused on the tribal hard-to-reach population, where evidence on TB-KAP is limited. The use of a tailored training manual developed and imparted by a multidisciplinary team ensures that the content is contextually relevant and scientifically robust. The inclusion of both students and women’s organisations from all the villages in the Senapati district enhances the community engagement and representation.

The limitation of the study is that since we have recruited students and women’s organisations volunteers only from the Senapati District, Manipur, the findings may not be generalizable beyond the specific socio-cultural context of the district, provided the unique demographic and cultural characteristics of the populations. Moreover, self-report measures of attitude and practice may have been influenced by social desirability bias. Also, since we have immediately assessed the training impact within a short time period, our ability to assess the sustainability of the impact remains limited and also the attrition of the volunteers during the study period may have affected the continuity, consistency, and intensity of the intervention delivery in some remote and hard-to-reach communities, despite ongoing efforts to recruit, train and retain the volunteers. Future studies should incorporate longer follow-up period and strategies to improve volunteers’ retention and evaluate the sustainability of community-based interventions. In addition, although the attitude scale included stigma-related items, a dedicated validated TB stigma instrument was not used; future studies should incorporate one to measure stigma more comprehensively.

## Conclusion

Our study demonstrated that focused (culturally appropriate) two-day hands-on training can substantially uplift TB-KAP and encourage favourable reported intentions even in the remotest tribal areas by equipping SAWOs with appropriate TB information. Our intervention effectively addressed the baseline knowledge deficits, enhanced perceptions and fostered favourable health seeking behaviour. Engaging volunteers from within the community, particularly SAWOs, offers a sustainable approach to strengthening awareness and advocacy for TB elimination. While the study findings are limited by their single district scope and short term assessment, they provide compelling evidence for integrating such a community based, culturally sensitive training model into the National TB program, with potential for adaptation in other tribal and underserved areas. As knowledge, attitudes and practices were assessed by self-report immediately after training and objective skills and field-level behaviour were not measured, longer-term follow-up and objective assessment are needed to confirm that these immediate gains translate into sustained practice.

## Supporting information

S1 Data TB-KAP analysis data. (XLS)
